# MHC Class I Molecules Exacerbate Viral Infection by Disrupting Type I Interferon Signaling

**DOI:** 10.1155/2019/5370706

**Published:** 2019-09-09

**Authors:** Simo Xia, Yijie Tao, Likun Cui, Yizhi Yu, Sheng Xu

**Affiliations:** National Key Laboratory of Medical Immunology, Institute of Immunology, Second Military Medical University, Shanghai 200433, China

## Abstract

MHC class I molecules are key in the presentation of antigen and initiation of adaptive CD8+ T cell responses. In addition to its classical activity, MHC I may possess nonclassical functions. We have previously identified a regulatory role of MHC I in TLR signaling and antibacterial immunity. However, its role in innate antiviral immunity remains unknown. In this study, we found a reduced viral load in MHC I-deficient macrophages that was independent of type I IFN production. Mechanically, MHC I mediated viral suppression by inhibiting the type I IFN signaling pathway, which depends on SHP2. Cross-linking MHC I at the membrane increased SHP2 activation and further suppressed STAT1 phosphorylation. Therefore, our data revealed an inhibitory role of MHC I in type I IFN response to viral infection and expanded our understanding of MHC I and antigen presentation.

## 1. Introduction

The innate immune system is the first line defense for viral infection. After recognition of certain pathogen-associated molecular patterns (PAMPs), diverse pattern recognition receptors (PRRs) trigger antiviral immune responses by inducing type I interferon (IFN) [1]. For RNA viruses, RIG-I and MDA5 are the main PRRs responsible for IFN production [2]. Type I IFN exerts its antiviral function by binding to its receptors and activating JAK-STAT signaling, which finally induces the expression of IFN-stimulated genes (ISGs) [3]. Both the production and downstream signaling of type I IFN are necessary for host innate antiviral immunity. Targeting type I IFN is the major mechanism employed by viruses to evade the host immune defense, and viruses have developed diverse strategies to circumvent the type I IFN system [4]. Although many regulators have been identified [5, 6], the details of fine-tuned IFN production and function remain unknown.

Major histocompatibility complex (MHC) class I molecules are among the primary two MHC molecules and are found on all nucleated cells. Their classical function is to display peptide fragments of endogenous antigens and present them to cytotoxic CD8 T cells [7, 8]. In vivo, MHC I is key for the selection of thymic CD8 T cells and is also involved in the education and tolerance of natural killer cells [9]. MHC I molecules are heterodimers composed of a heavy chain and a *β*2 microglobulin, and *β*_2_m is essential for the stable expression of MHC I on a cell membrane. The heavy chain is composed of two extracellular Ig-like domains and an intracellular domain. In contrast to MHC class II molecules, MHC I molecules have a longer intracellular tail with approximately 40 amino acids, including a tyrosine site [10]. As tyrosine phosphorylation is a key posttranscriptional modification involved in signal transduction cascades [11], MHC I molecules are expected to have nonclassical functions in signal transduction.

Although MHC I molecules always function as ligands, reverse signaling was demonstrated two decades ago and plays important roles in cell apoptosis, activation, or function. Cross-linking MHC I on T cells triggers Lck, Zap70, and PLC*γ*1 activation, which leads to T cell activation [12, 13] or apoptosis [14]. Cross-linking MHC I on NK cells segregates MHC I from NK cell synapse, induces intracellular phosphotyrosines, and inhibits NK cell function [15]. MHC I can also initiate intracellular signals in endothelial cells and smooth muscle cells, eliciting cell proliferation in synergy with growth factors [16]. In malignant tumor, anti-MHC I or anti-*β*_2_m antibodies can specifically induce tumor cell apoptosis [17, 18]. We have previously demonstrated an inhibitory role of MHC I in TLR signaling in myeloid cells, which facilitated bacterial infection [19]. However, the role of MHC I on viral infection remains unknown.

Here, we reported that the lack of MHC I significantly suppressed viral replication in macrophages independent of IFN production, which depended on the disrupted IFN signaling pathway. Mechanically, after viral infection, MHC I enhanced SHP2 activation, which suppressed STAT1 phosphorylation and led to reduced ISG production. Our data revealed an inhibitory role of MHC I in type I IFN signaling during viral infection and expanded our understanding of MHC I function and antigen presentation.

## 2. Materials and Methods

### 2.1. Mice

C57BL/6 mice were from Joint Ventures Sipper BK Experimental Animals (Shanghai). Mice deficient in H-2Kb and H-2Db were from Taconic Farms and termed as MHC I-deficient mice. All animals were bred in specific pathogen-free conditions, and all animal experiments were in accordance with the National Institute of Health Guide for the Care and Use of Laboratory Animals, with the approval of the Scientific Investigation Board of the Second Military Medical University, Shanghai, China.

### 2.2. Cell Culture

Bone marrow-derived macrophages (BMDMs) were generated as previously described. Briefly, bone marrow cells were isolated from the femur and tibia and cultured in 10% RPMI1640 with 20% L929 cell-conditioned medium as a source of GM-CSF. Three to four days after seeding, the supernatants were removed and attached cells were further cultured with conditional medium for additional 3-5 days. The remaining cells were collected and used as macrophages.

### 2.3. Viral Infection and Viral Quantification

VSV virus was amplified in 293T cells, and mice were infected at 10^8^ PFU per gram of body weight intraperitoneally. Cultured supernatants or tissue homogenates were serially diluted and added into monolayer BHK21 cells. At the end point, the amount of virus required to kill 50% of infected cells was determined as 50% Tissue Culture Infective Dose (TCID_50_).

### 2.4. Bronchoalveolar Lavage Fluid (BALF) Collection

For BALF, the trachea was cannulated and lavaged with 1 ml PBS for two times. Collected withdrawing samples were centrifuged at 3,000 rpm for 5 min, and the cell pellets were resuspended in PBS and counted as infiltrating lymphocytes.

### 2.5. Histopathology

The lung tissues were fixed in 10% formalin, embedded in paraffin, cut, and stained with hematoxylin and eosin. The histopathologic inflammation score of lung tissues was evaluated by a pathologist blinded to the experimental design. Lung inflammatory changes were graded using a semiquantitative scoring system based on the following parameters: peribronchiolar and bronchial infiltrates, bronchiolar and bronchial luminal exudates, perivascular infiltrates, parenchymal pneumonia, and edema, as previously described [20]. Each parameter was graded on a scale of 0–4: 0, absent; 1, slight; 2, mild; 3, moderate; and 4, severe.

### 2.6. Real-Time PCR

RNA were extracted with an RNA purification kit (Fastagen, Shanghai) and reverse transcribed with a PrimeScript RT-PCR kit (Takara, Japan). The primers for Ifnb, Ifn4a Isg15, Isg54, Oas, and Mx1 were from the PrimerStar website. The following are the primers of VSV: forward 5′-ACG GCG TAC TTC CAG ATG G-3′ and reverse 5′-CTC GGT TCA AGA TCC AGG T-3′. The mRNA expression was done with an SYBR Premix Ex Taq qPCR kit (Takara) by LightCycler (Roche) and analyzed with the *ΔΔ*T method. Data were normalized with *β*-actin expression.

### 2.7. ELISA Assay

Cytokines in the supernatant of cell culture were collected and diluted as needed and analyzed using a mouse IFN-*β* ELISA kit (PBL Biomedical Laboratories) according to the manufacturer's instructions.

### 2.8. Flow Cytometry and Intracellular Staining

For intracellular cytokine staining, macrophages were stimulated *in vitro* with VSV for 8 hours, and protein transport inhibitor brefeldin A was added during the last 4 hours. Cells were collected and fixed with Fixation & Permeabilization Buffer (BioLegend). Then, cells were stained with intracellular IFN-*β* with anti-mouse IFN-*β* mAb-biotin (BioLegend), followed by secondary streptavidin-PE staining. Flow cytometry analyses were performed using FACSVantage (Becton Dickinson). Data were analyzed by FACSDiva.

### 2.9. Immunoprecipitation and Immunoblot

Cells were lysed with cell lysis buffer (CST, USA), supplemented with protease inhibitor cocktail (Calbiochem). Protein concentration was determined with BCA assay (Pierce), and equivalent proteins were loaded for western blotting or immunoprecipitation. Immunoblot was performed with anti-STAT1 (9172, CST), anti-p-SHP2 (3703, CST), anti-p-STAT1 (D4A7, CST), anti-p-JAK1 (3331, CST), and anti-p-Tyr (9416, CST) antibodies. And anti-H2Kb (MHC I, AF6-88.5) was from BioLegend.

### 2.10. Gene Overexpression and Silencing

MHC I molecule H-2Kb was transfected with JetPEI reagents (PolyPlus, France), and 24 hours later, overexpression was confirmed by western blot. The siRNA targeting Shp2 was from Dharmacon and transfected with an INTERFERin reagent (PolyPlus) according to a standard protocol. The silencing efficiency was confirmed with western blot analysis.

### 2.11. Statistical Analysis

The statistical significance between two groups was determined by Student's *t*-test. For the comparison of more than 2 groups, one-way ANOVA was adopted, and Fisher's exact LSD test was used for the intergroup comparison. For two independent variables, two-way ANOVA was adopted for statistical analysis, and Tukey's multiple comparison method was used for the intergroup comparison. Probability values less than 0.05 were considered to be statistically significant.

## 3. Results

### 3.1. MHC I Promotes Viral Replication Independent of Suppressing Type I IFN Production

**Figure 1:**
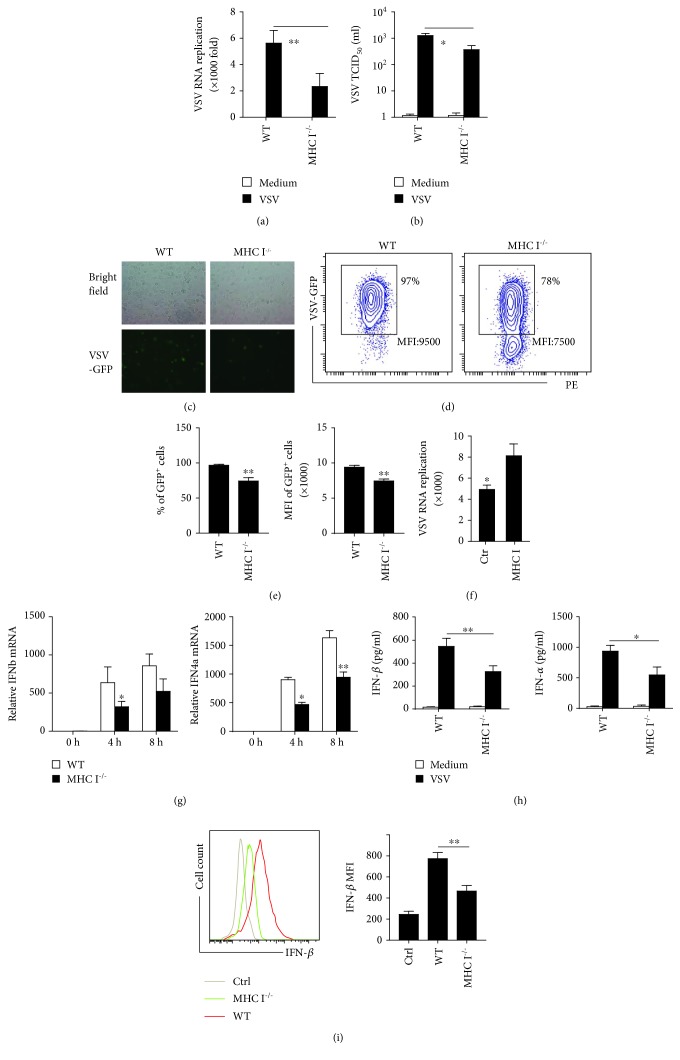
MHC I promotes viral infection independent of type I IFN. (a) VSV RNA replication in WT and MHC I^−/−^ macrophages stimulated with VSV for 6 h, as determined by real-time qPCR analysis. (b) Detection of VSV viral load by TCID_50_ in the supernatant from WT and MHC I^−/−^ macrophages. (c) VSV-GFP replication in WT or MHC I^−/−^ macrophages after 12 h and visualized by fluorescence microscopy. (d) Flow cytometry analysis of WT and MHC I^−/−^ macrophages infected with VSV-GFP after 16 h. (e) Percentages and FITC MFI of GFP^+^ cells in (d). (f) VSV RNA replication in WT macrophages overexpressed with MHC I or control vectors. (g) Relative type I IFN mRNA expression in WT and MHC I^−/−^ macrophages at indicated times post infection. (h) Type I IFN in the supernatant of VSV-infected WT and MHC I^−/−^ macrophages 24 h later, analyzed by ELISA assay. (i) Intracellular IFN-*β* staining of WT and MHC I^−/−^ macrophages post VSV infection (left) and statistical MFI of IFN-*β* (right). Data are the mean ± SD of at least three independent experiments. Two-way ANOVA was adopted for statistical analysis in (a), (g), and (h). One-way ANOVA was adopted for statistical analysis in (i). Student's *t*-test was adopted for analysis in (e) and (f). ^∗^*p* < 0.05 and ^∗∗^*p* < 0.01.

Our previous data revealed that MHC I molecules not only are key to adaptive CD8 T cell responses but are also involved in the fine tune of innate inflammatory cytokine production and antibacterial infection [19]. To examine whether MHC I is involved in innate antiviral immune responses, we first infected macrophages from MHC I-deficient mice and littermate control mice with VSV. Deficiency of MHC I caused significant decreased replication of VSV RNA in macrophages (Figure 1(a)). The VSV TCID_50_ in the supernatants also confirmed a reduced VSV load in MHC I-deficient macrophages (Figure 1(b)). In addition, a VSV-GFP infection model was used to directly determine the viral load in infected cells. A fluorescence plot also confirmed a lower virus load in MHC I-deficient macrophages (Figure 1(c)). To quantify the data, the macrophages were further collected for flow cytometry analysis (Figure 1(d)). Both the percentage and the mean fluorescence intensity (MFI) of GFP-positive cells were decreased in MHC I^−/−^ macrophages (Figure 1(e)). To further investigate the role of MHC I in viral infection, we overexpressed MHC I in macrophages. As expected, overexpression of MHC I promoted VSV replication (Figure 1(f)). These data demonstrate a promoting function of MHC I in viral infection.

Type I IFNs are the key antiviral innate cytokines. More type I IFN production would lead to reduced viral load in infected cells. To gain insight into the mechanism by which MHC I deficiency ameliorated viral load, type I IFN production was determined. Instead of upregulating these innate antiviral cytokines, MHC I deficiency reduced IFN-*α* and IFN-*β* mRNA levels in macrophages, (Figure 1(g)), which was confirmed by ELISA assay (Figure 1(h)). The cytokines in the supernatant by ELISA assay reflect the effect of cytokine secretion minus conception. To exclude reduced type I IFNs caused by more conception, we detected the IFN-*β* production by intracellular staining (Figure 1(i)). The flow cytometry data also revealed reduced intracellular IFN-*β* production in MHC I^−/−^ macrophages. These data indicated that decreased viral load in MHC I-deficient macrophages cannot be attributed to the upregulation of type I IFN production. In contrast, decreased viral load may be the reason for the reduced type I IFN production.

### 3.2. MHC I Inhibited Type I IFN Signaling and ISG Induction

**Figure 2:**
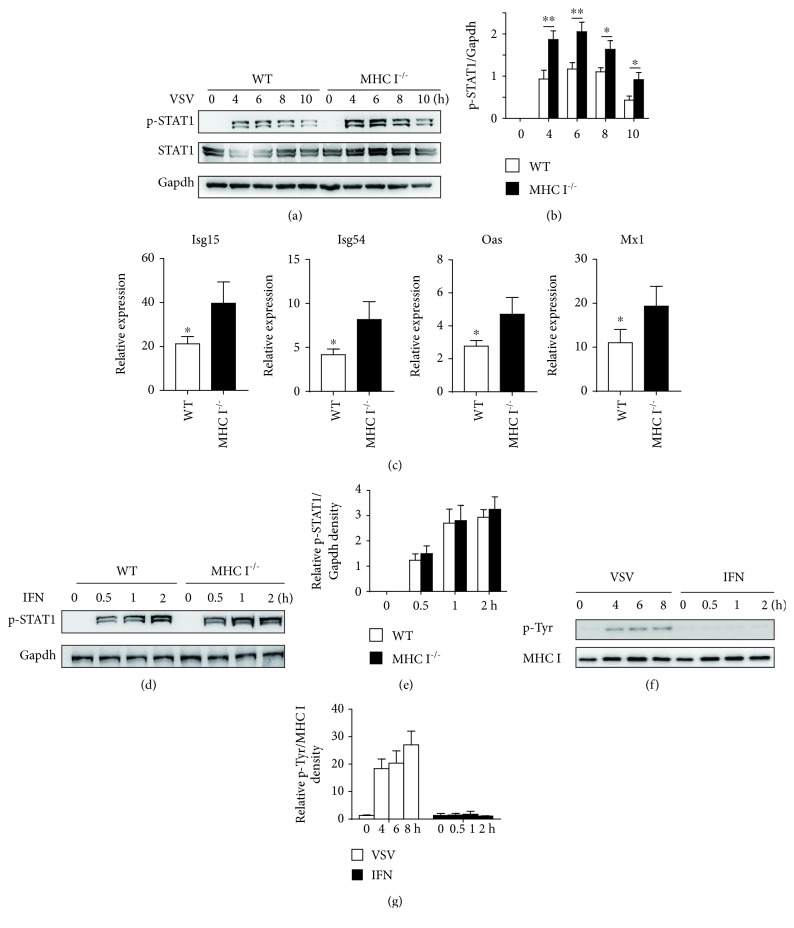
MHC I inhibited IFN downstream signaling. (a) Western blot analysis of STAT1 phosphorylation in WT and MHC I^−/−^ macrophages post VSV infection. (b) Relative density of the blot in (a). (c) Representative ISG expression in WT and MHC I^−/−^ macrophages post VSV infection. (d) Western blot analysis of STAT1 activation in WT and MHC I^−/−^ macrophages treated with IFN-*β* for the indicated times. (e) Relative density of the blot in (d). (f) Phosphorylation of MHC I after coimmunoprecipitation with anti-H2Kb antibody visualized by immunoblot with anti-p-Tyr. (g) Relative density of the blot in (f). Data are the mean ± SD of at least three independent experiments. Two-way ANOVA was adopted for statistical analysis in (b), (e), and (g). Unpaired Student's *t*-test was adopted for statistical analysis in (c). ^∗^*p* < 0.05.

As type I IFNs bind receptors to exert its antiviral effect, we next examined whether MHC I deficiency influenced type I IFN downstream signaling. MHC I deficiency caused increased STAT1 phosphorylation in macrophages, without influencing STAT1 expression (Figures 2(a) and 2(b)). The antiviral effect of type I IFN mainly depends on ISG expression. We also found elevated ISG15, ISG54, OAS, and Mx1 expressions in MHC I-deficient macrophages (Figure 2(c)). These data strongly indicated that MHC I deficiency could promote type I IFN signaling and its antiviral activity.

To elucidate the role of MHC I in IFN signaling, we directly stimulated macrophages with IFN-*β* in vitro. However, we found no significant difference of STAT1 activation in MHC I-deficient macrophages compared with that in WT macrophages (Figures 2(d) and 2(e)). As MHC I intracellular tyrosine phosphorylation was necessary for its function in TLR signaling [19], we speculated that IFN stimulation alone may not induce MHC I phosphorylation. Figures 2(f) and 2(g) confirm that VSV infection induced significant phosphorylation of MHC I, while IFN stimulation did not have a similar effect. These data indicated a regulatory effect of MHC I in type I IFN signaling, which is dependent on its tyrosine phosphorylation.

### 3.3. MHC I Suppressed IFN Signaling through SHP2 Activation

**Figure 3:**
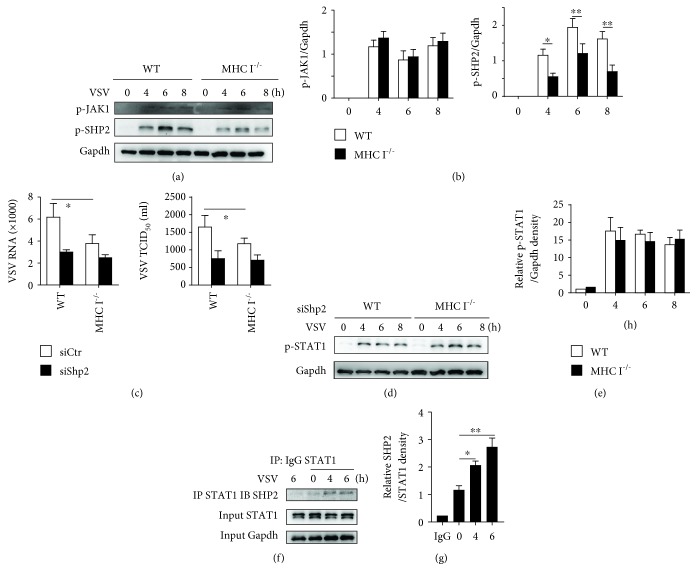
MHC I suppressed IFN signaling through SHP2. (a) Western blot analysis of JAK1 and SHP2 phosphorylation in WT and MHC I^−/−^ macrophages at the indicated times post VSV infection. (b) Relative density of the blot in (a) from 3 independent experiments. (c) VSV RNA replication (left) and TCID_50_ (right) in WT and MHC I^−/−^ macrophages with Shp2 silenced before infection. (d) Western blot analysis of STAT1 activation in WT and MHC I^−/−^ macrophages with Shp2 silenced before infection. (e) Relative density of the blot in (d). (f) Immunoblot assay of SHP2 and STAT1 post coimmunoprecipitation with anti-STAT1. (g) Relative density of the blot in (f). Data are the mean ± SD of at least three independent experiments. Two-way ANOVA was adopted for statistical analysis in (b), (c), and (e). One-way ANOVA was adopted for statistical analysis in (g). ^∗^*p* < 0.05.

After binding to the IFN receptor, the JAK-STAT pathway was activated and finally led to ISG production [21]. Though p-STAT1 was upregulated in MHC I-deficient macrophages, the activation of JAK1 was not significantly altered in the MHC I-deficient macrophages compared with that in WT cells (Figures 3(a) and 3(b)). These data suggested that MHC I may target STAT1 to regulate the type I IFN signaling pathway.

We previously revealed that during TLR stimulation phosphorylated MHC I sustained SHP2 activation. As MHC I was also phosphorylated post VSV infection, we wondered whether SHP2 was also involved in the regulation of IFN signaling during viral infection. We found obvious SHP2 activation at the indicated times post viral infection, which was suppressed in MHC I-deficient macrophages (Figures 3(a) and 3(b)). Knocking down SHP2 expression reduced the viral load in both the WT and MHC I-deficient macrophages (Figure 3(c)). In addition, SHP2 knockdown abrogated the reduction of viral load in MHC I-deficient macrophages compared with that in WT macrophages (Figure 3(c)). Silencing Shp2 also abrogated the difference in STAT1 activation between WT and MHC I-deficient cells (Figures 3(d) and 3(e)). In addition, SHP2 was found to interact with STAT1 after VSV infection (Figures 3(f) and 3(g)). These data strongly suggested that SHP2 is necessary for MHC I-mediated suppression of IFN signaling.

### 3.4. The Biological Relevance of MHC I Regulation of Type I IFN Signaling

**Figure 4:**
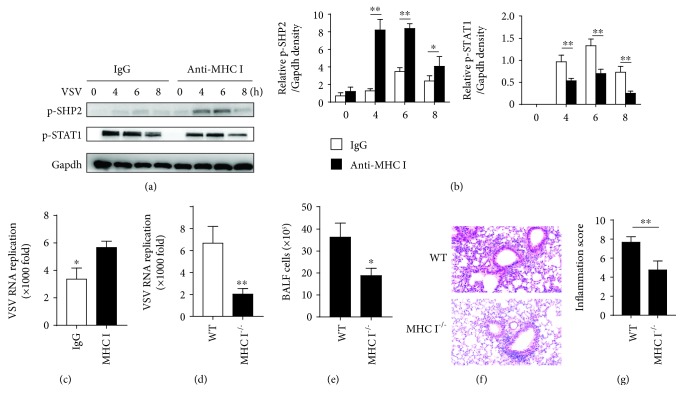
MHC I promotes viral replication during the innate phase of infection in vivo. (a) Macrophages were cross-linked with anti-MHC I antibodies, and the activation levels of SHP2 and STAT1 were determined by western blot assay at indicated times post VSV infection. (b) Relative density of the blot in (a). (c) Relative VSV replication in macrophages in (a). (d) WT and MHC I^−/−^ mice were infected i.p. with VSV; viral load in the lung was calculated 18 h later. (e) BALF cell numbers in the lung from WT and MHC I^−/−^ mice infected with VSV. (f) H&E analysis of the VSV-infected lung from mice infected with VSV (200x). (g) Inflammation score of the lung damage in (f). Data are the mean ± SD of at least three independent experiments. Two-way ANOVA was adopted for statistical analysis in (b). Unpaired Student's *t* test was adopted for statistical analysis in (c), (d), and (e). ^∗^*p* < 0.05 and ^∗∗^*p* < 0.01.

We next wanted to reveal the biological and pathologic relevance of MHC I-mediated IFN signaling suppression. The main function of MHC I is presenting an antigen to TCRs to form immune synapse. In the immune synapse, pMHC-TCR aggregated into clusters and thus amplified the signaling. To mimic this cluster formation, we cross-linked MHC I molecules with anti-MHC I antibodies in vitro. The cross-linking increased SHP2 phosphorylation, inhibited STAT1 activation after VSV infection (Figures 4(a) and 4(b)) and exacerbated viral replication in macrophages (Figure 4(c)).

We further infected MHC I-deficient mice and littermate control mice with VSV. One day post infection, the viral titers in the lung were significantly lower in MHC I-deficient mice than they were in the littermate controls (Figure 4(d)). There were reduced infiltrating lymphocyte in BALF from MHC I^−/−^ mice compared with that in WT mice (Figure 4(e)). H&E analysis of the infected lung also revealed less extensive lymphocyte infiltration in peribronchiolar and perivascular areas in MHC I-deficient mice (Figure 4(f)). A semiquantitative analysis of the inflammation score of the inflammatory lung showed less inflammation in MHC I^−/−^ mice, compared with that in WT mice (Figure 4(g)). These data indicated the greater resistance of MHC I-deficient mice to viral infection during the early phase of infection. Thus, all these data have suggested a suppressive role of MHC I in type I IFN signaling, which was dependent on SHP2 activation and STAT1 dephosphorylation.

## 4. Discussion

MHC I belongs to the Ig superfamily, and most of the cell surface proteins in this family are engaged in cellular recognition and intercellular signaling. The primary function of MHC I molecules is to work as ligands, providing antigen signals for CD8 T cells. The nonclassical function of MHC I molecules was revealed more than two decades ago, and nonclassical function has been observed in T cells, B cells, NK cells, myeloid cells, endothelial cells, and tumor cells [13, 14, 22]. In T cells and B cells, cross-linking MHC I activated lck/zap70 and lyn/syk, respectively, and induced T/B cell activation, proliferation, or apoptosis, which depends on the specificity of antibody [12, 14, 23]. Specifically, in malignant tumors, especially in myeloma, anti-MHC I antibody selectively induced tumor cell apoptosis, by activating Lyn and PLC*γ*2 to upregulate proapoptotic Bad and Bax expression [18]. Here, we reported a nonclassical function of MHC I in macrophages: suppression of type I IFN signaling to impair innate antiviral immunity. In vivo data confirmed that MHC I-deficient mice were more resistant than WT mice in the very early time of viral infection.

Type I IFNs are the key innate antiviral cytokines and include IFN-*α*, IFN-*β*, IFN-*κ*, IFN-*δ*, IFN-*ε*, IFN-*τ*, IFN-*ω*, and IFN-*ζ*, with IFN-*α* and IFN-*β* as the most well defined types [24]. Type I IFN is induced when microbial products are sensed by PRRs and functions in an autocrine or paracrine manner. After binding to its receptors IFNAR1 and IFNAR2, type I IFN activates JAK1 and TYK2. Phosphorylation of IFNAR by these kinases recruits STAT proteins (STAT1 and STAT2), resulting in their phosphorylation, dimerization, and nuclear translocation [21]. These transcription factors bind to IFN-stimulated response element (ISRE) sequences to activate antiviral ISG transcription. The regulation of type I IFN production has been studied extensively [25], and there are also molecules which fine tune the downstream IFN signal pathway [21]. However, although MHC I is key to antiviral adaptive immunity, its role in innate antiviral immune regulation and type I IFN signaling remains undetermined, and our study may extend our understanding of MHC I.

MHC I deficiency reduced viral replication in macrophages, but did not increase type I IFN production. These data suggested that the reduced viral load cannot be attributed to more type I IFN production. In contrast, the reduced IFN secretion may be the result of reduced viral burden in MHC I-deficient macrophages. Increased STAT1 activation and ISG production in MHC I-deficient cells confirmed our speculation that MHC I impaired type I IFN downstream signal transduction. To determine the mechanism by which MHC I molecules inhibit type I IFN signaling, we first examined whether the interaction occurred at the STAT1 level or the upstream level (IFNAR and JAK1). Considering the activation of JAK1 was not different in WT and MHC I-deficient cells during viral infection, we concluded that STAT1 may be the target of MHC I.

Our previous study suggested that MHC I may recruit Fps and then activate SHP2 in myeloid cells [19]. Previous data have also revealed that SHP2 can regulate type I IFN signal transduction [26, 27]. Without SHP2, MHC I deficiency had no significance on viral replication, suggesting an indispensable role of SHP2 in MHC I function during VSV infection. Our study showed that MHC I inhibited IFN signaling through SHP2 activation and that SHP2 may bind directly to STAT1 to reduce STAT1 phosphorylation.

Considering the mechanism by which MHC I activates SHP2, the “open conformer” theory of MHC I molecules has been proposed [28]. A pool of MHC I at the membrane can dissociate from the antigen peptide, becoming the open MHC I conformers. These open conformers can associate with other receptors and possess hidden functions. The formation of open MHC I conformers depends on phosphorylation of its intracellular Tyr320 [29]. In our study, the suppression function of MHC I was also dependent on its tyrosine phosphorylation, suggesting that an open conformer may be needed for its inhibitory function. Sole type I IFN stimulation did not induce tyrosine phosphorylation, thus abrogating the suppressive function of MHC I.

In conclusion, our study has demonstrated a suppressive role of MHC I molecules in type I IFN signaling. Our findings provided new insight into the fine tune of antiviral type I IFN immune responses and indicated a nonclassical function of MHC I in antiviral responses.

## Data Availability

The data used to support the findings of this study are available from the corresponding authors upon request.
